# Near-Infrared Spectroscopy Machine-Learning Spectral Analysis Tool for Blueberries (*Vaccinium corymbosum*) Cultivar Discrimination

**DOI:** 10.3390/foods14081428

**Published:** 2025-04-21

**Authors:** Pedro Ribeiro, Maria Inês Barbosa, Clara Sousa, Pedro Miguel Rodrigues

**Affiliations:** CBQF—Centro de Biotecnologia e Química Fina—Laboratório Associado, Escola Superior de Biotecnologia, Universidade Católica Portuguesa, Rua de Diogo Botelho 1327, 4169-005 Porto, Portugal; s-pmsbribeiro@ucp.pt (P.R.); mibarbosa@ucp.pt (M.I.B.); cssousa@ucp.pt (C.S.)

**Keywords:** blueberries, infrared spectroscopy, cultivar, taxonomy, vaccinium, machine learning

## Abstract

*Vaccinium corymbosum* is one of the main sources of commercialized blueberries across the world. This species has a large number of distinct cultivars, leading to significantly different berries characteristics such as size, sweetness, production rate, and growing season. In this context, accurate cultivar discrimination is of significant relevance, and currently, it is mostly performed through berries examination. In this work, we developed a method to discriminate 19 cultivars from the *V. corymbosum* species through their leaves’ near-infrared spectra. Spectra were acquired from fresh blueberry leaves collected from two geographic regions and across three seasons. Machine-learning-based models, selected from a pool of 10 classifiers based on their discrimination power under a twofold stratified cross-validation process, were trained/tested with 1 to 20 components obtained by the application of data dimensionality reduction (DDR) techniques (dictionary learning, factor analysis, fast individual component analysis, and principal component analysis) to different near-infrared (NIR) spectra regions’ data, to either analyze a single spectral region and season or combine spectral regions and/or seasons for each side of the blueberry leaf. The percentage of correct cultivar discrimination ranged from 52.27 to 100%, with the best spectral results obtained with the adaxial side of the leaves in the fall (100% Accuracy) and the abaxial side of the leaves in the winter (100% Accuracy); the fast ICA DDR technique was present in 83.33% of the best analyses (five out of six); and the LinearSVC was present in 66.67% (four out six best analyses). The results obtained in this work denote that near-infrared spectroscopy is a suitable and accurate technique for *V. corymbosum* cultivar discrimination.

## 1. Introduction

The *Vaccinium* genus from the Ericaceae family encompasses about 450 species, widespread mostly in the Northern Hemisphere [1,2,3]. Some of its species are of particular relevance due to their highly appreciated small berries. *V. corymbosum*, one of the main sources of commercialized blueberries, is among them. This species encompasses a high number of cultivars possessing different characteristics such as production rates and growing seasons, fruit sweetness, and size, and requires distinct edaphoclimatic conditions for their optimum development (such as temperature, soil pH, and daylight hours), among others. Therefore, accurate discrimination of such cultivars is of the utmost relevance either in agricultural practices or in the food industry. Currently, blueberry cultivar identification is mostly performed by farmers based on visual inspection of the phenotypic characteristics of the berries [4]. However, due to their high similarity, there is a high rate of misidentification. Indeed, to the best of our knowledge, only two attempts for blueberry cultivar identification have been performed: one based on germplasm characterization [5], and the other on EST-PCR markers [6]. Despite presenting a high potential for subspecies identification, these techniques are expensive and time-consuming. Therefore, it is important to find reliable and cost-effective alternative techniques. Spectroscopic-based techniques, due to their advantages of low cost, speed, absence of sample preparation, real-time results, and reagent-free operation, could be an interesting alternative and are gathering strong acceptance in the typing context, mainly at the genus and species levels [7,8,9,10]. However, the infra-species level has been barely explored, particularly in plants with only a few works published [11,12,13,14,15,16,17]. Among these, Kim S. et al. [11] demonstrated that Fourier-transform infrared (FT-IR) spectroscopy combined with multivariate analysis allows rapid and effective discrimination between commercial strawberry cultivars based on their metabolic profiles. Similarly, a 2012 study [12] showed that Fourier-transform mid-infrared (FT-MIR) spectroscopy and chemometric analysis could effectively distinguish five Tunisian olive cultivars using the chemical composition of their leaves. Moura L. et al. [13], in 2015, used spectral reflectance analysis with nonlinear regression and principal component analysis to automatically classify different lettuce cultivars. That same year, Li X. et al. [14] demonstrated that near-infrared (NIR) spectroscopy combined with pattern recognition methods such as backpropagation neural network (BPNN) and least-squares support vector machines (LS-SVMs) effectively identified various *Pummelo* cultivars. More recently, Kasampalis D. et al. [15] used visible/near-infrared (Vis/NIR) spectral reflectance data from potato tuber skin to assess postharvest freshness and distinguish between cultivars. Yilmaz-Düezyaman H. et al. [17] showed that NIRS can predict oxidative stability and differentiate extra virgin olive oil cultivars, while Difante G. et al. [16] applied NIR spectroscopy combined with machine-learning (ML) models to differentiate *Panicum maximum* cultivars based on leaf spectral reflectance. Despite previous studies, further research is necessary as NIR spectroscopy has not yet been applied to the identification of *V. corymbosum* cultivars.

The present study explores the potential of NIR spectroscopy combined with ML tools to discriminate 19 cultivars of *V. corymbosum* based on the infrared spectra of their fresh leaves (adaxial and abaxial sides). For that, the spectra were collected from two geographic regions across three different seasons, and a pool of 10 ML-based approaches are evaluated for discriminating *V. corymbosum* blueberry cultivars using NIR spectra across different seasons and leaf sides (abaxial and adaxial). Thus, the classifiers are fed with NIR data selected from the ANOVA F-value method (*p* < 0.05) and reduced to 1 to 20 components using four data dimensionality reduction (DDR) techniques: dictionary learning (DL), factor analysis (FA), fast independent component analysis (fast ICA), and principal component analysis (PCA). It should be noted that we discarded at the beginning the possibility of using deep-learning models instead of ML models, as they often require large amounts of labeled data and significant computational resources, and they can be seen as “black boxes”, making it difficult to interpret the results and understand the underlying decision-making process.

## 2. Materials and Methods

### 2.1. Vaccinium Cultivar Leaves

Different numbers of adult leaves of *V. corymbosum* belonging to 19 distinct cultivars, fully healthy, 3.7 to 7.2 cm long and 1.1 to 2.6 cm wide, and exposed to sunlight, were collected in 2 distinct regions in the north of Portugal during 3 seasons of 2017:

- Spring: 44 plants—44 leaves, 1 per plant;
- Fall: 22 plants—22 leaves, 1 per plant;
- Winter: 32 plants—32 leaves, 1 per plant.

Leaves from Region 1 (R1) were collected in three seasons: spring, fall, and winter, while leaves from the second region (R2) were collected only in spring. Details about the samples are presented in Table 1. Eng. Paulo Lúcio Gomes, an expert on the area from Bagas de Portugal (https://www.bagasdeportugal.pt/, accessed on 11 April 2025), identified the species.

### 2.2. Infrared Spectra Acquisition

NIR spectra were acquired from fresh leaves. Spectra were collected from two distinct leaves per plant on both the adaxial and abaxial surfaces, in two separate spots. A total of eight spectra per plant (2 × 2 × 2) were obtained, avoiding rib leaves. The NIR spectra were acquired using a Fourier-transform near-infrared spectrometer (FTLA 2000, ABB, Québec, QC, Canada) equipped with an indium–gallium–arsenide (InGaAs) detector in diffuse reflectance mode. Each spectrum was the result of an average of 64 scans with a resolution of 8 cm^−1^, within the wavenumber interval of 10,000 to 4000 cm^−1^. Bomen-Grams software (version 7, ABB, Québec, QC, Canada) was used to control the equipment.

### 2.3. Data Analysis and Prediction

**Figure 1:**
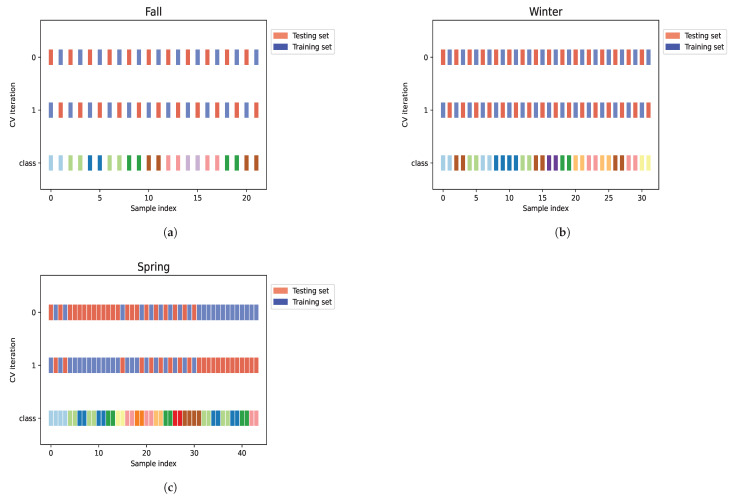
The stratified k-fold approach employed for data classification using ML models, per season: (**a**) fall, (**b**) winter, and (**c**) spring.

The infrared spectra were imported into Python (version 3.9.21, developed by the Python Software Foundation, Wilmington, DE, USA) and adjusted by applying the z-score normalization method. Five different regions (Region I: 9920–7275 cm^−1^; Region II: 7274–6314 cm^−1^, Region III: 6313–5390 cm^−1^, Region IV: 5389–4924 cm^−1^, and Region V: 4923–4073 cm^−1^) and the whole NIR spectra were evaluated to find information for maximizing the performance of ML models and to check if there is a region that provides better information for discrimination. For that, in those regions and in the whole spectrum, the ANOVA F-value method was applied for feature selection (*p* < 0.05) to select frequency bins ranging from 30 to the maximum bins available per region of analysis (687 for Region I, 249 for Region II, 240 for Region III, 120 for Region IV, 221 for Region V, and 1517 for whole spectra), with increments of 10 bins, in an iterative process. After that, the models were trained and tested with varying numbers of components using four DDR: DL, FA, fast ICA, and PCA, ranging from 1 to 20, with the rest of the hyperparameters being set to default, to guarantee that the discrimination power to classify the *V. corymbosum* species was optimized. In addition to that, 10 pre-designed scikit-learn ML models were used for the classification task. Table 2 shows the classifiers used and their configuration. The choice of the best model considered their discrimination power using a 2-fold stratified cross-validation process (see Figure 1) to ensure the same proportion of class labels per fold and to maximize the performance of the model [18]. To validate the performance of each model, the classification report with values of Accuracy, Precision, Recall, *F1-score*, and area under the receiver operating characteristic (ROC) curve (AUC) was obtained.

The *Accuracy* was calculated using the equation below and represents the proportion of correctly classified classes relative to all cases [19]:

(1) $$Accuracy=\frac{TP+TN}{TP+TN+FP+FN}\times 100\%$$

where TP, TN, FP, and FN correspond to true positives, true negatives, false positives, and false negatives, respectively [20].

*Precision* represents the ratio of correctly classified positive cases to the total cases predicted as positive [21]:

(2) $$Precision=\frac{TP}{TP+FP}\times 100\%$$

*Recall* represents the ratio of correctly predicted positive cases to the total number of actual positive cases [21]:

(3) $$Recall=\frac{TP}{TP+FN}\times 100\%$$

The *F1-score* is the harmonic mean of *Recall* and *Precision* [22]:

(4) $$F1-\mathrm{Score}=\frac{2\times Precision\times Recall}{Precision+Recall}\times 100\%$$

*AUC* evaluates the model’s capability to distinguish between positive and negative classes by analyzing true positive (TP) and false positive (FP) rates across different thresholds. *AUC* values range from 0 to 1, where 1 represents a perfect classifier, and 0.5 indicates random classification [23].

**Figure 2:**
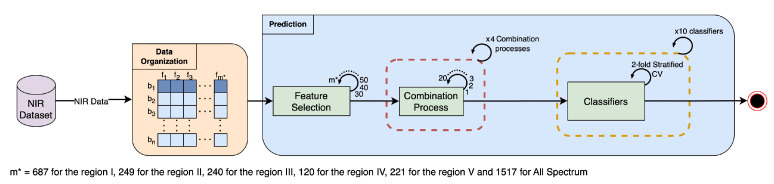
Data processing and prediction workflow.

A summary of the data analysis and prediction methodology is illustrated in Figure 2.

## 3. Results

### 3.1. Spectral Analysis Results

**Figure 3:**
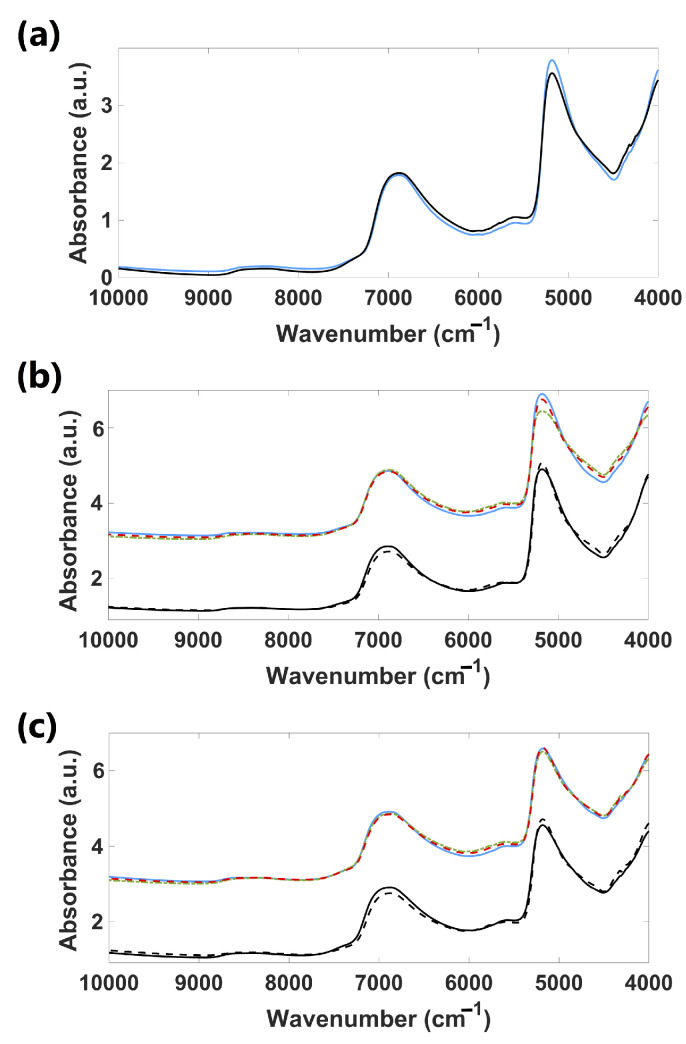
*Vaccinium* leaves mean NIR spectra (Chandler cultivar) of (**a**) fresh adaxial (—) and abaxial (—) surfaces; (**b**) fresh adaxial and (**c**) fresh abaxial of: spring (—), fall (– ·), and winter (— —) leaves from R1 and spring leaves from regions R1 (—) and R2 (— —). **Note:** Just for illustrative purposes, we separated the NIR spectra by magnitude to effectively show the differences to the naked eye. This separation was necessary because the spectral frequency bins significantly overlap along the wavenumber, making it difficult to distinguish the spectra visually. That is why in the present figure, NIR spectra of different cultivars exhibit higher values compared to those associated with regions.

**Figure 4:**
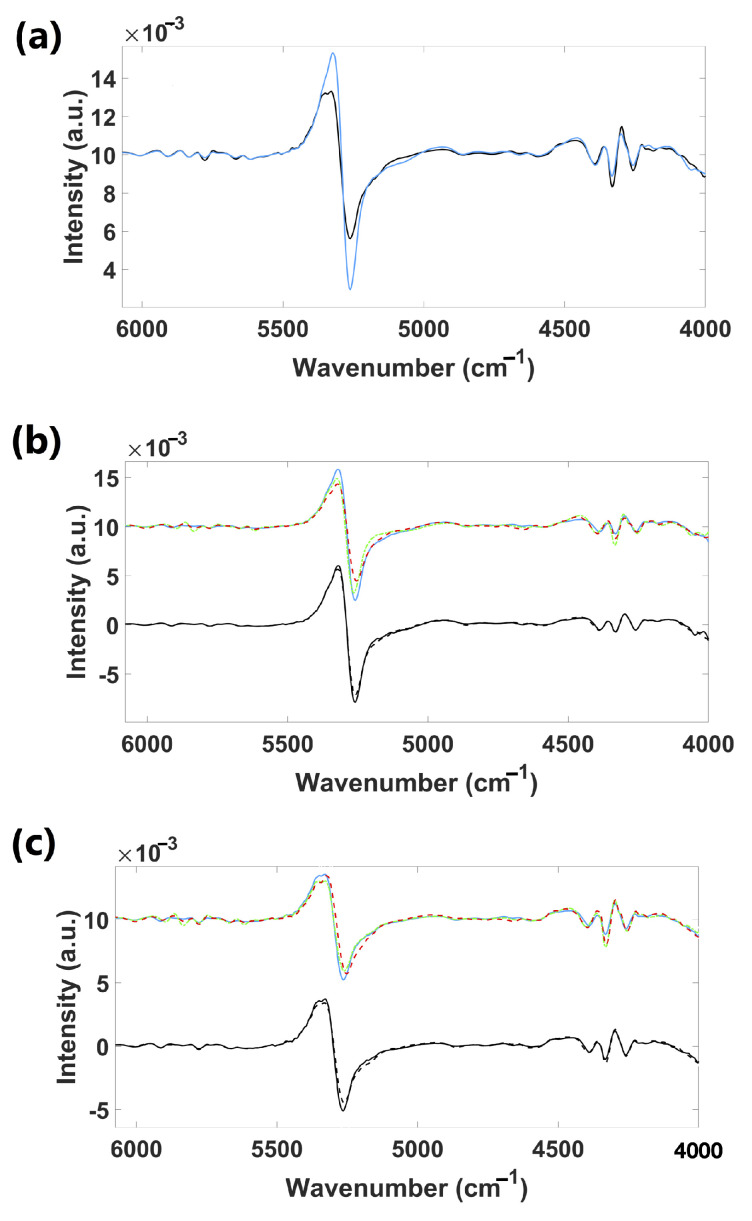
*Vaccinium* leaves second derivative [SavGol filter (15, 2, 2)] mean NIR spectra (Chandler cultivar) of (**a**) fresh adaxial (—) and abaxial (—) surfaces; (**b**) fresh adaxial and (**c**) fresh abaxial of: spring (—), fall (– ·), and winter (— —) leaves from R1 and spring leaves from regions R1 (—) and R2 (— —). **Note:** (1) We zoomed into the 6100–4000 cm^−1^ region as it provided more differences within the second derivative mean NIR spectra. (2) Just for illustrative purposes, we separated the NIR spectra by magnitude to effectively show the differences to the naked eye. This separation was necessary because the spectral frequency bins significantly overlap along the wavenumber, making it difficult to distinguish the spectra visually. That is why in the present figure, NIR spectra of different cultivars exhibit higher values compared to those associated with regions.

Figure 3a presents the mean NIR spectra of all the samples (cultivars) of the fresh leaves (adaxial and abaxial sides), and Figure 4a, the corresponding derivatives. The spectra of both sides are quite similar, with only slight differences. Broad bands at 5200 and 7000 cm^−1^ are associated with the O–H combination and the first O–H overtones of water, respectively [24]. Vibration bands around 5800–5650 cm^−1^, 4900–4500 cm^−1^, and 4300–4200 cm^−1^ were observed. Bands within 5800–5650 cm^−1^ can be attributed to C–H vibrations in the first overtone region, connected to cellulose [25]. Bands from 4900 to 4500 cm^−1^ are associated with N–H and N–H plus C–H combinations, related to starch, pectin, and cellulose [25]. Bands around 4300–4200 cm^−1^ are located in the C–H plus C–C combination band regions, linked to cellulose and proteins [26].

Figure 3b,c present the mean NIR spectra for the Chandler cultivar (adaxial and abaxial sides of fresh leaves), and Figure 4b,c, the corresponding derivatives. Spectra of three different seasons (spring, fall, and winter) obtained from region R1 and spectra from spring leaves of R2 were presented. Spectra showed great similarity, especially those from the abaxial side of fresh leaves. Few or no naked-eye differences were observed between spectra from different seasons except for regions from 5900 to 5300 cm^−1^ (C–H first overtone, C=O second overtone vibrations, and O–H combinations associated with lignin, cellulose, sugars, starch, and proteins [26]), and 4900 to 4200 cm^−1^ (N–H combinations, N–H + C–H combinations, C–H + C–H combinations, and C–H + C–C combinations related to starch, proteins, pectin, and cellulose [25,26]) of the adaxial side spectra. Fall and winter spectra seem to have slightly more intense vibration bands in these regions. The spring leaves spectra from R1 and R2 showed slight differences in the region from 4900 to 4200 cm^−1^.

### 3.2. Machine-Learning Classification Results

**Figure 5:**
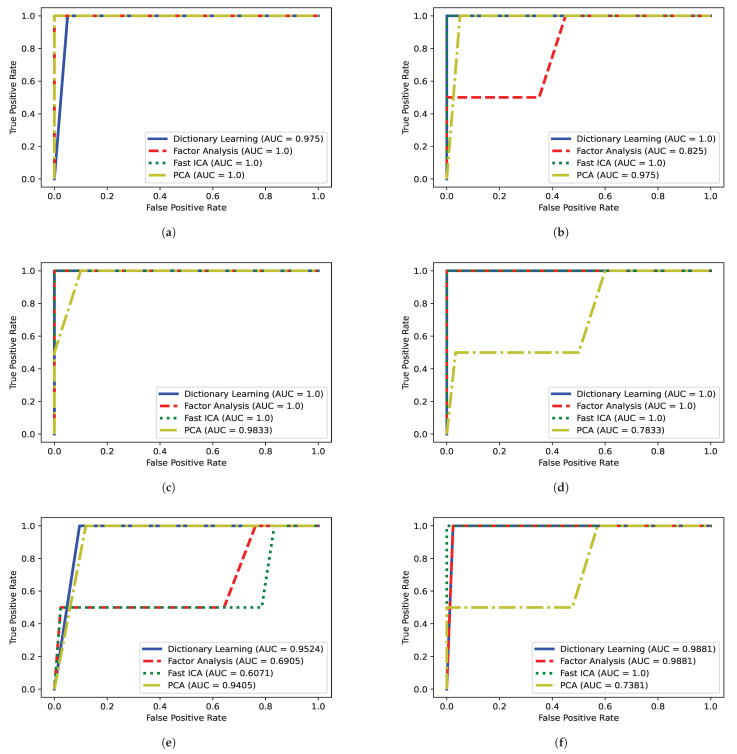
*AUC* ROC curves obtained with different DDR processes for each season and both sides of the leaves. (**a**) ROC curve of the adaxial side of the leaf during fall; (**b**) ROC curve of the abaxial side of the leaf during fall; (**c**) ROC curve of the adaxial side of the leaf during winter; (**d**) ROC curve of the abaxial side of the leaf during winter; (**e**) ROC curve of the adaxial side of the leaf during spring; (**f**) ROC curve of the abaxial side of the leaf during spring.

Figure 5 illustrates the ROC curves derived from the discrimination analysis of *V. corymbosum* cultivars, per season and leaf region. The curves were generated from models trained with NIR data dimensionality reductive by four different techniques: DL, FA, fast ICA, and PCA.

In Table 3, the Accuracy and AUC results for the best models fed with data from different spectral regions (I: 9920–7275 cm^−1^, II: 7274–6314 cm^−1^, III: 6313–5390 cm^−1^, IV: 5389–4924 cm^−1^, and V: 4923–4073 cm^−1^) and from the entire spectrum are presented, categorized by season and leaf side. Highlighted in green are the best results achieved for each season and leaf side, based on the highest Accuracy and AUC values.

Table 4 provides a summary of the overall best model’s performance results highlighted in green in Table 3 per season, and adaxial–abaxial leaf face. This summary includes Accuracy, Precision, Recall, *F1-score*, and AUC, considering the number of components and the number of frequency bins (features).

**Figure 6:**
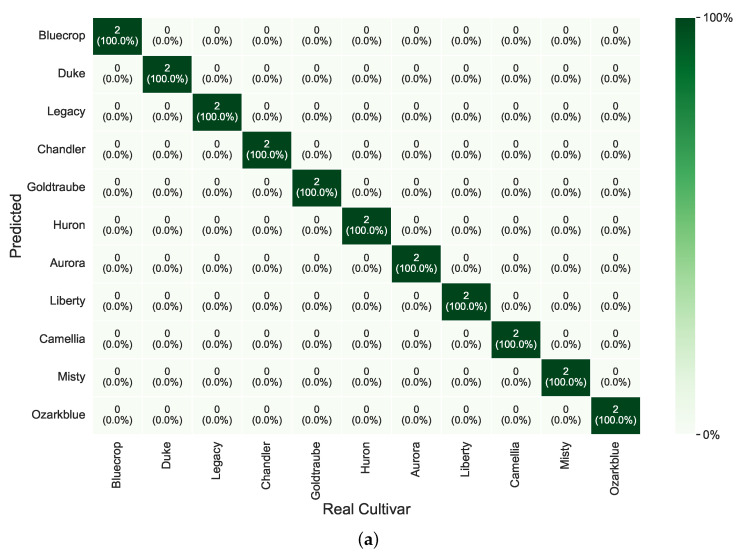

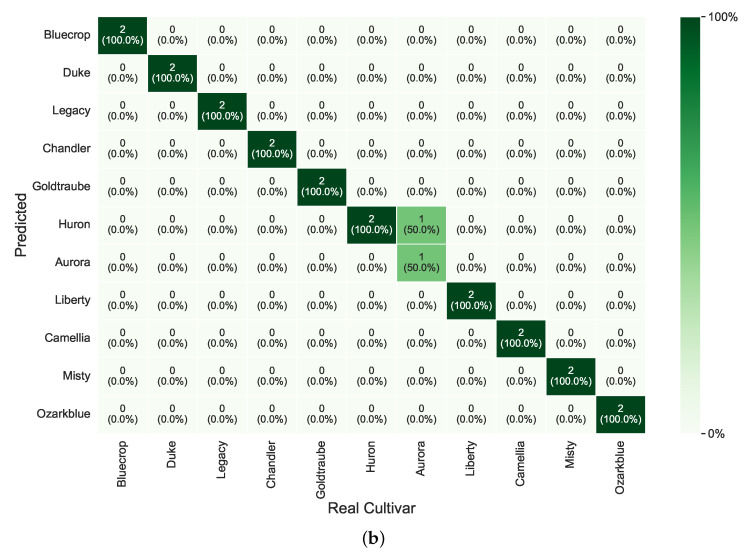
Confusion matrices with prediction Accuracy in percentage (%) for the best overall results during fall. (**a**) Adaxial side of the leaf. (**b**) Abaxial side of the leaf.

**Figure 7:**
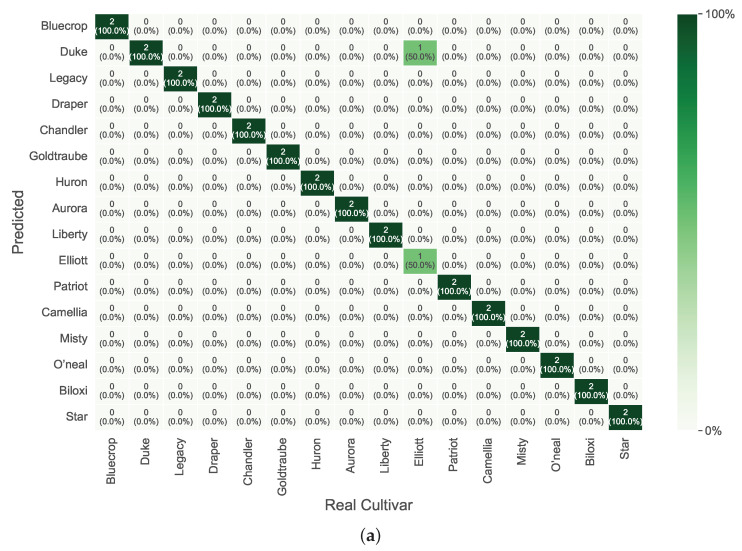

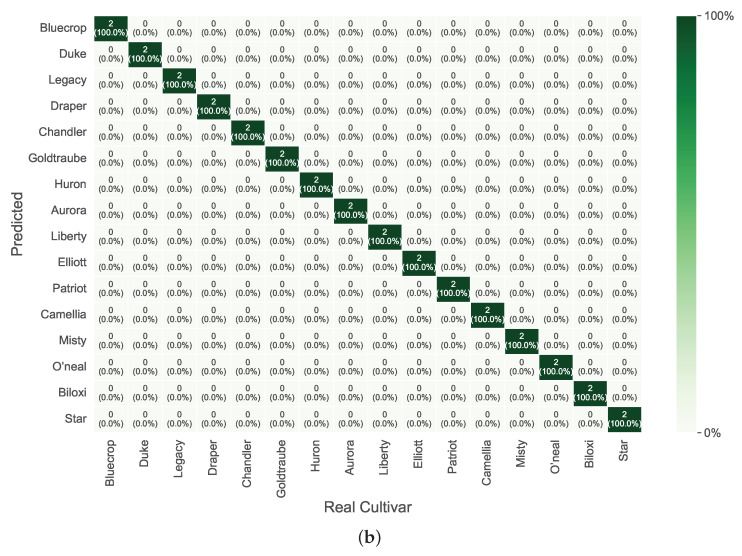
Confusion matrices with prediction Accuracy in percentage (%) for the best overall results during winter. (**a**) Adaxial side of the leaf. (**b**) Abaxial side of the leaf.

**Figure 8:**
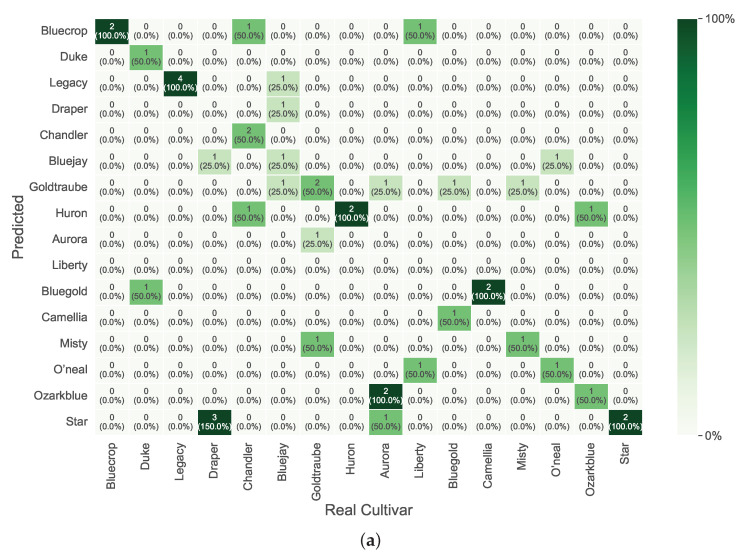

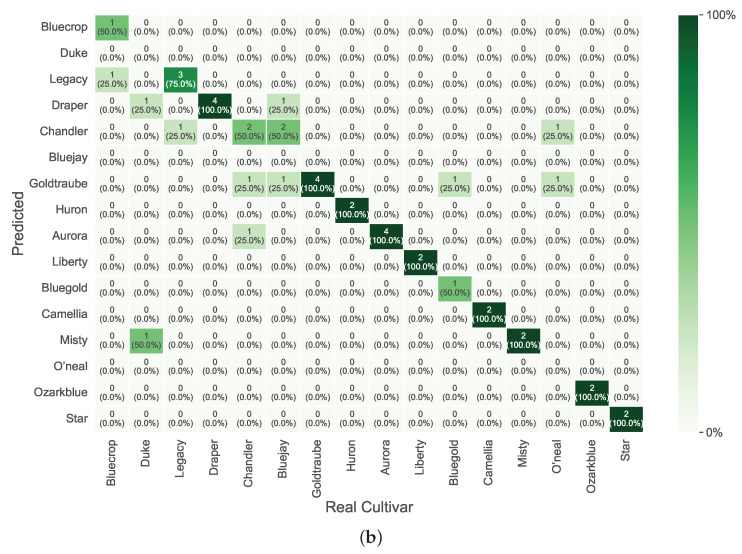
Confusion matrices with prediction Accuracy in percentage (%) for the best overall results during spring. (**a**) Adaxial side of the leaf. (**b**) Abaxial side of the leaf.

Considering the best overall results, the confusion matrices for the discrimination process of *V. corymbosum* cultivar are presented in Figure 6,Figure 7,Figure 8, per season and leaf side.

## 4. Discussion

Near- and mid-infrared spectroscopy are being used for plant typing at different taxonomic levels [7,11,12,13,14], disease detection [27], antioxidant properties [28], and storage duration estimations [29], among others. Of the different plant constituents commonly explored, leaves are the ones that yield the best results. Despite the success of these techniques for typing purposes, their ability for cultivar discrimination has barely been explored, with few published studies [11,14], and has never been tested to discriminate blueberry cultivars. In this context, this work aimed to evaluate the ability of NIR spectroscopy to discriminate *V. corymbosum* cultivars from two different geographical regions and different seasons.

### 4.1. Choosing the Best DDR Method for Each Season and Leaf Side Using the AUC ROC Curves

Looking at the results presented in Figure 5, it can be seen that the fast ICA was the best overall DDR process, with 83.33% (five out of six) figures indicating an *AUC* of 1. The DL and the FA reached an *AUC* value of 1 in 50% (three out of six) of the cases. Lastly, the PCA DDR process achieved an *AUC* of 1 in 16.67% (one out of six) of the possible cases. With these results in mind, we see that the fast ICA is the best DDR technique choice for the majority of the cases, except when classifying the leaves’ adaxial side in spring, where DL proved to be a superior technique for DDR. This can be explained due to our data probably following a non-Gaussian distribution in which fast ICA performs better. In the case of PCA and FA, these techniques have an advantage in Gaussian distributions [30]. The DL possibly did not show a better result because it requires careful fine-tuning [31].

### 4.2. Choosing the Best Spectral Region for Each Season and Leaf Side Using AUC and Accuracy

In Table 3, we present the best spectral region results for the optimal DDR process, categorized by season and leaf side. The selection of the best region was based on the AUC and Accuracy metrics. In the fall, when we used the leaves’ adaxial side, we can see that the best results came from the spectral region III, with the AUC and Accuracy obtaining the results of 1 and 100%, respectively. In the case of the leaves’ abaxial side for the same season, the best region was region III, with an AUC of 1 and an Accuracy of 95.45. For the spring, the best results for the leaves’ abaxial side came from the all spectrum region, with an AUC of 0.619 and an Accuracy of 52.27%. The best results from the abaxial leaves in spring came from region III, with an AUC and Accuracy of 1 and 70.45%, respectively. In the case of the winter season, the adaxial of the leaves obtained the best results in region V, reaching 1 of AUC and a 96.88% Accuracy. For the abaxial of the leaves in the case of the winter season, the best results were 1 of AUC, and 100% of Accuracy illustrated in region V, as well. Globally, we can see a clear dominance of region III, corresponding to 50% (three out of six) of the cases, followed by region V with 33.33% (two out of six), and last was region II, which represented 16.67% (1 out of 6).

### 4.3. Analysis of the Best Overall Results

In Table 4, we see that the fall and winter seasons yielded the best results. The Adaxial_Fall and Abaxial_Winter achieved 100% in *Accuracy*, *Precision*, *Recall*, and *F1-score*, with the *AUC* reaching 1. The Adaxial_Spring had an *Accuracy* of 52.27%, a *Precision* of 38.54%, a *Recall* of 48.44%, an *F1-score* of 40.63%, and an *AUC* of 0.619.

The Adaxial_Winter achieved an *Accuracy*, *Precision*, *Recall*, *F1-score*, and *AUC* of 96.88%, 95.31%, 96.88%, 95.83%, and 1, respectively. The Abaxial_Fall demonstrated an *Accuracy* of 95.45%, a *Precision* of 93.18%, a *Recall* of 95.45%, an *F1-score* of 93.94%, and an *AUC* of 1. The Abaxial_Spring had an *Accuracy* of 70.45%, a *Precision* of 61.46%, a *Recall* of 70.31%, an *F1-score* of 64.69%, and an *AUC* of 1.

The best classifier was the LinearSVC, representing 66.67% (four out of six) of the models. A possible reason why a simple classifier, such as LinearSVC, achieves the best results in most comparison groups is the size of the dataset. More complex classifiers tend to introduce higher variance, which is disadvantageous for small datasets [32].

Globally, better results were achieved with fall and winter leaves. Spring leaves yielded poorer cultivar identification rates, which could be related to their higher water and chlorophyll contents. Water and chlorophyll are, particularly in spring leaves, major components and could mask spectra, making other components (discriminant ones) less visible to the infrared light [33].

### 4.4. Analysis of Confusion Matrices Related to the Best Overall Results

Figure 6 shows the confusion matrices for the leaves’ sides in the fall. We can see that the adaxial side did not have any difficulty discriminating between classes. However, on the abaxial side, there is a slight difficulty with the *Aurora* and *Huran* classes.

Figure 7 illustrates the confusion matrices for the leaves’ sides in winter. The adaxial side presented some difficulty in correctly discriminating the *Elliott* class, which was often predicted as the *Duke* class. In contrast, the abaxial side showed clear discrimination between all classes.

Figure 8 presents the confusion matrices for the leaves’ sides in spring. The adaxial side shows a significant difficulty in correctly discriminating the classes, with only the *Bluecrop*, *Legacy*, *Huron*, and *Star* classes being correctly classified. On the abaxial side, there is a small improvement compared to the adaxial side in the same season. However, there is still some difficulty in correctly classifying the *O’neal*, *Bluegold*, *Bluejay*, *Chandler*, *Legacy*, *Duke*, and *Bluecrop* classes. Overall, the cultivar discrimination success cannot be linked to a specific leaf side (adaxial or abaxial). The best results seem to be randomly achieved with one leaf side.

## 5. Conclusions

Overall, this study demonstrated the potential of NIR spectroscopy for infra-species typing and established a reliable method for blueberry cultivar identification, addressing a clear gap in the literature. The proposal model’s discrimination Accuracy of cultivars with air-dried and fresh leaf spectra ranged from 52.27% to 100%, with the highest spectral performance achieved using the adaxial side of the leaves in fall and winter, both with 100% Accuracy in spectral regions III and V, respectively. It is important to highlight the higher number of cultivars tested (19 different cultivars) correctly classified with a low number of LVs. To the best of our knowledge, no prior studies have reported spectral recognition results for *V. corymbosum* leaves that are optimized in fall and winter; therefore, a comparison between studies was not possible. This aspect should be explored in future research to better understand how seasonal factors influence model performance.

Additionally, our proposed methodological approach showed that a calibration model must be used to carefully identify cultivars from different geographic regions or seasons, enabling an accurate identification of blueberry cultivars without the need for berries on the plant. Based on these findings, a calibrated and validated portable NIR device can allow farmers to conduct on-site cultivar identification and real-time decision making without the need for expert support. To enhance the current study, future research will focus on optimizing the hyperparameters of classifiers and DDR techniques. Additionally, the incorporation of more samples will facilitate the application of advanced deep-learning-based data analysis, thereby improving the generalizability of the results.

## Figures and Tables

**Table 1:** *V. corymbosum* leaves collection included in this work.

Species	Blueberry Type	Cultivar	Spring (30th May)		Fall (1st September)	Winter (1st December)
			R1	R2	R1	R1
*V. corymbosum*	Northern	Bluecrop	2	2	2	2
		Duke	2	2	2	2
		Legacy	2	2	2	2
		Draper	2	2		2
		Chandler	2	2	2	2
		Bluejay	2			
		Goldtraube	2		2	2
		Huron	2		2	2
		Aurora	2		2	2
		Liberty	2		2	2
		Elliott				2
		Patriot				2
		Bluegold		2		
	Southern	Camellia	2	2	2	2
		Misty	2		2	2
		O’neal	2			2
		Ozarkblue	2		2	
		Biloxi				2
		Star	2			2

**Table 2:** 10 Scikit-learn ML classifier configurations.

Classifier	Hyperparameters
AdaBoostClassifier (AdaBoost)	Default parameters
BaggingClassifier (BaggC)	Default parameters
DecisionTreeClassifier (DeTreeC)	max_depth: 5
GaussianNB (GauNB)	Default parameters
KNearestNeighborsClassifier (KNN)	Default parameters
LinearDiscriminantAnalysis (LinDis)	Default parameters
LinearSVC (LinSVC)	random_state = 0
LogisticRegression (LogReg)	solver: “lbfgs”
QuadraticDiscriminantAnalysis (QuadDis)	Default parameters
Support-vector Machines (SVC)	γ: “auto”, probability = 1

**Table 3:** *Accuracy* and *AUC* results for each spectra region and for the whole spectrum (per season and leaves side).

	DDR	I (9920–7275 ${\mathrm{cm}}^{-1}$)		II (7274–6314 ${\mathrm{cm}}^{-1}$)		III (6313–5390 ${\mathrm{cm}}^{-1}$)		IV (5389–4924 ${\mathrm{cm}}^{-1}$)		V (4923–4073 ${\mathrm{cm}}^{-1}$)		All Spectrum (9920–4073 ${\mathrm{cm}}^{-1}$)	
	Process	*AUC*	*Accuracy*	*AUC*	*Accuracy*	*AUC*	*Accuracy*	*AUC*	*Accuracy*	*AUC*	*Accuracy*	*AUC*	*Accuracy*
Adaxial_Fall	Fast ICA	0.8	81.82	0.975	77.28	1	100	1	68.19	1	90.9	1	81.82
Adaxial_Spring	DL	0.5833	40.91	0.9524	43.18	0.6429	50	0.619	40.91	0.5714	45.45	0.619	52.27
Adaxial_Winter	Fast ICA	0.9833	75	0.9833	84.38	1	93.75	0.9833	78.13	1	96.88	0.96667	84.38
Abaxial_Fall	Fast ICA	0.9	77.27	0.5	77.27	1	95.45	0.725	81.82	1	86.36	0.975	90.91
Abaxial_Spring	Fast ICA	0.9524	34.09	1	52.27	1	70.45	0.9881	50	1	59.09	0.9881	54.55
Abaxial_Winter	Fast ICA	1	71.88	1	84.38	1	93.75	0.75	87.5	1	100	1	87.5

**Note:** Highlighted in green are the best results achieved per season and leaf side, based on the highest Accuracy and AUC values.

**Table 4:** Best overall results for the best region highlighted in Table 3.

	Spectral Region	DDR Process	# of Components	# of Features	Classifier	*Accuracy*	*Precision*	*Recall*	*F1-Score*	*AUC*
Adaxial_Fall	III	Fast ICA	12	140	LinSVC	100	100	100	100	1
Adaxial_Spring	All Spectrum	DL	15	330	LinSVC	52.27	38.54	48.44	40.63	0.619
Adaxial_Winter	V	Fast ICA	14	120	LogReg	96.88	95.31	96.88	95.83	1
Abaxial_Fall	III	Fast ICA	10	110	LogReg	95.45	93.18	95.45	93.94	1
Abaxial_Spring	III	Fast ICA	13	190	LinSVC	70.45	61.46	70.31	64.69	1
Abaxial_Winter	V	Fast ICA	15	190	LinSVC	100	100	100	100	1

## Data Availability

The data presented in this study are available on request from the corresponding author due to privacy reasons.
